# Agronomic Evaluation and Chemical Characterization of *Salvia lavandulifolia* Vahl. over 3 Consecutive Years Cultivated Under Harsh Climatic Conditions in Southeast Spain

**DOI:** 10.3390/plants13213022

**Published:** 2024-10-29

**Authors:** Gustavo J. Cáceres-Cevallos, María Quílez, Gonzalo Ortiz de Elguea-Culebras, Enrique Melero-Bravo, Raúl Sánchez-Vioque, María J. Jordán

**Affiliations:** 1 Research Group on Rainfed Agriculture for Rural Development, Department of Rural Development, Oenology and Sustainable Agriculture, Murcia Institute of Agri-Food and Environmental Research (IMIDA), La Alberca de las Torres, 30150 Murcia, Spain; gustavoj.caceres@carm.es (G.J.C.-C.); maria.quilez@carm.es (M.Q.); 2 Instituto Regional de Investigación y Desarrollo Agroalimentario y Forestal de Castilla La Mancha (IRIAF), CIAF de Albaladejito, Carretera Toledo-Cuenca Km 174, 16194 Cuenca, Spain; gonzaloo@jccm.es (G.O.d.E.-C.); emelerob@jccm.es (E.M.-B.); rsanchezvioque@gmail.com (R.S.-V.)

**Keywords:** *Salvia lavandulifolia* Vahl., agronomic yield, essential oil, phenolic profile, antioxidant activity, rainfed crop

## Abstract

The cultivation of *Salvia lavandulifolia*, Spanish sage, makes an important contribution to the economy of many rural areas in Southeastern Spain. This aromatic plant species is characterized by high intraspecific variability, which makes the selection process for the establishment of homogeneous crops difficult. Additionally, imminent climate change threatens to reduce its production, especially when cultivated in drylands. Therefore, to guarantee the continued production of this type of sage, it is essential to study its agronomic behavior and production quality. For this, clones from four ecotypes were cultivated for three years, assessing changes in their biomass production, essential oil yield and quality, and phenolic fraction, as well as the corresponding antioxidant activity. The results suggest that essential oil yield is genetically predetermined, greater biomass not being associated with higher quantities of essential oil. Weather conditions affected both essential oil and phenolic fraction secondary metabolism. Under very harsh conditions, Spanish sage produces higher concentrations of camphor and 1,-8-cineole along with luteolin-7-O-glucoside, and lithospermic, rosmarinic, and salvianolic A acids in its phenolic fraction. The synthesis of these components helps the species to withstand the hot and dry conditions typical of southeast Spain.

## 1. Introduction

*Salvia lavandulifolia* Vahl., commonly known as Spanish sage, is a herbaceous perennial native to the Iberian Peninsula that can be found in the western Mediterranean, at altitudes from 300 to 1000 m above sea level [1]. Its adaptability to the semi-arid Mediterranean climate conditions, as well as its pharmacological and medicinal properties, explain why this aromatic plant is attracting interest as a potential alternative rainfed crop, with high commercial value [2].

The exploitation of this species is mainly focused on the extraction of essential oil (EO). Nevertheless, EO production seems to show high intraspecific variability with the geographic origin of the plants [2,3,4]. Specifically, it has been found that wild Spanish sage grown in southern and central Spain contains relatively high levels of 1,8-cineole, while that grown in the north contains more camphor and its biosynthetic precursor borneol [5]. In relation to this, Dosoky et al. [6] conducted an extensive study of cultivated Spanish sage from 2016 to 2021 in southern Spain. Chromatographic analysis of the EO allowed the identification of a chemotype characterized by 1,8-cineole (24.3–34.0%) and camphor (23.5–28.8%) as major components of the volatile fraction. On the other hand, Sánchez-Vioque et al. [7] analyzed the EO composition of macropropagated Spanish sage plants collected from 12 wild populations and established in an experimental plot. Based on the mean relative concentrations of EO components, they observed a chemotype defined by camphor (15.3%), 1,8-cineole (15.0%), α-pinene (11.3%), β-pinene (8.5%), and limonene (7.5%).

One of the industry’s main concerns in the extraction of aromatic and medicinal plant EOs is the quantity of residues generated, though it is well known that these materials could serve as an important source of bioactive compounds with a range of applications [8]. According to Santana-Meridas et al. [9], approximately 100 kg of solid waste is generated for every 1 kg of EO produced. Applied to the production of EOs from aromatic plants in the Region of Murcia (southeast Spain), estimated at 230 tons in 2020 [10], this implies the generation of some 203,000 tons of residues.

Among the numerous health properties described for Spanish sage extracts [11,12,13,14], perhaps the most notable is a potent antioxidant activity, related to its polyphenolic content [15]. However, as occurs with the EO, the polyphenolic profile in this species varies depending on the geographic origin of the plants [16]. In line with this, rosmarinic acid, salvigenin, apigenin-7-O-glucoside, and luteolin-7-O-glucuronide were identified as major components in the methanolic extracts of *Salvia lavandulifolia* from different wild populations in Spain [1,17], whereas rosmarinic acid, quercetin rhamnose, diosmetin-rutinoside, catechin dimer, and gallocatechin were the major compounds quantified from wild populations in Morocco [15].

Taking this intraspecific variability into account, among the main obstacles to the commercial cultivation of this species are the lack of plant material selection and knowledge of changes in agronomic performance over time, as these issues make it difficult to safeguard the quality of the production. As stated above, the observed chemical intraspecific variability depends on the geographic origin, but it is also known that abiotic stress influences the component ratios that define the EO chemotype [2,7,18]. These results were obtained in experimental crops based on the cultivation of non-preselected plants. Thus, the usual variability of wild populations was transferred to the cultivation areas. Given all this, to ensure good production, there is a need to select and propagate individual plants with high productivity and quality, and to investigate changes in their agronomic characteristics from year to year [19].

In this context, the main objective of our study was to conduct an agronomic assessment of four preselected ecotypes of *Salvia lavandulifolia* Vahl. (Sa1–Sa4) cultivated under the semi-arid conditions of southeast Spain over three consecutive years. To the best of our knowledge, under these harsh edaphoclimatic conditions, no studies have been conducted related to the stability and behavior of this potential crop over time, assessing the agronomic and EO yields, EO composition, quantitative polyphenolic profile, and the antioxidant activity of polyphenolic compounds identified.

## 2. Results

### 2.1. Biomass Production and Essential Oil Yield

**Figure 1:**
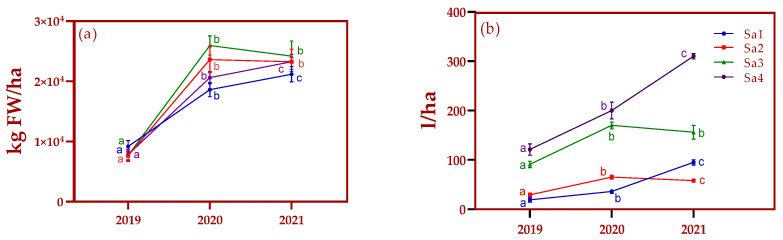
Measurements of (**a**) agronomic and (**b**) essential oil yield in *Salvia lavandulifolia* Vahl. in three consecutive years. Different letters indicate a significant difference between years at *p* < 0.05.

An agronomic evaluation, considering EO yield and phytomass growth, was performed in four ecotypes of *Salvia lavandulifolia*. Figure 1a shows that production tripled from the first to second year for most of the ecotypes, and then remained at similar levels in the third year of cultivation. The exception was Sa1, for which production only doubled; this was the ecotype with the lowest biomass yield. At first glance, considering the level of annual precipitation in 2020 (528 mm), the strong increase in phytomass production could be attributed to the larger amount of rainwater received by plants. On the other hand, this argument is undermined by the level of production achieved in 2021 despite the low rainfall (396 mm).

Regarding the EO (Figure 1b), significant increases were also observed in the second year of cultivation, with the yield doubling compared to the first harvest in 2019. Further, the volumes of EO from Sa1 and Sa4 increased from the second to the third year, and this increase did not reflect their biomass growth. Overall, after three years of cultivation, EO yield reached its highest value in two ecotypes, while it fell slightly with respect to the second year in the other two.

### 2.2. Essential Oil Composition

The GC-MS analysis allowed the identification of 36 major components, which accounted for 95–100% of the volatile fraction of *Salvia lavandulifolia* EO (Table 1). The main components identified were camphor and 1,8-cineole, followed by camphene and α-pinene, compounds that define the chemotype (based on the order of abundance of the main constituents) in these sage ecotypes.

After micropropagation and establishment of the preselected ecotypes in the experimental area, the analysis of EO volatile profile after the first year of cultivation (Table 1) revealed that the chemotypes were very similar to those described in the same individuals when preselected in the wild and stored in the IMIDA germplasm bank (data reported in Section 4). Nonetheless, differences were detected in the relative concentration of the components. In particular, regarding camphor, the relative concentration increased after multiplication and cultivation, especially in Sa3, doubling from 17 to 32%. Analyzing 1,8-cineole, a mixed pattern was observed, the concentration of this ether decreasing in two of the ecotypes (Sa1 and Sa3), increasing in one (Sa4), and remaining stable in another (Sa2). It is also worth mentioning the presence of borneol in Sa1 and Sa2, the relative concentration of this terpene alcohol increasing significantly in the volatile profile of these plants after cultivation. In contrast, the relative concentration of the terpene hydrocarbons α-pinene and camphene significantly decreased.

Overall, after three consecutive years of cultivation, changes were observed in all crops in the relative concentration of the components that define the chemotype, especially camphor, 1,8-cineole, and borneol. First, levels of camphor decreased significantly over the three years of cultivation in the case of Sa1, reaching a reduction of close to 32%. Similar behavior was observed in Sa2 and Sa3, the relative concentrations of camphor decreasing significantly from the first to the second year, by percentages that ranged between 19 and 29%, though levels of this ketone remained steady during the third year. Nevertheless, in Sa4, the reduction in camphor was less marked (13%) and significant differences were only found between the first and the third year of the study.

Concerning 1,8-cineole, the second most abundant volatile component identified in Sa2, Sa3, and Sa4, pronounced reductions in concentration were seen from the first to the second year of cultivation. These corresponded to losses of 81%, 69%, and 47%, respectively, and although there were slight increases between the second and third year, the levels did not reach those recorded at the beginning of the experiment.

The pattern for borneol, the third most abundant component identified in the volatile profile of all four salvia ecotypes under study, differed from those of camphor and cineole. Specifically, significant increases in relative concentration were detected between the first and second years in Sa3 and Sa4, but there were losses in the third year.

In relation to the monoterpene hydrocarbon fraction, some components, including α-pinene, sabinene, β-pinene, *p*-cymene, (*E*)-β-ocimene, γ-terpinene, and α-terpinolene, showed similar behavior, their concentration increasing progressively from the first to the third year of the study. This pattern was also observed for the terpene alcohol terpinen-4-ol and the sesquiterpenes β-caryophyllene and α-humulene (in Sa2 and Sa3). The relative concentrations of spathulenol and caryophyllene oxide increased from the first to the second year of cultivation but fell during the third year of cultivation. Lastly, in the cases of α-terpineol, linalool, linalyl acetate, and terpinyl acetate (the last three detected at a high concentration in Sa3), a significant drop in concentration was observed from year one to year two, followed by a recovery in year three.

### 2.3. Phenolic Profile

Regarding phenolic profiles, HPLC-DAD analysis allowed the identification of 19 phenolic compounds, with rosmarinic acid, salvianolic acid A, salvigenin, and luteolin-7-O-glucuronide being the most abundant phenols in the first year of cultivation in all ecotypes (Table 2). As described for the EO volatile profile, the phenolic quantitative composition also varied over the three years of cultivation.

Although the response was ecotype dependent, in the second year, in which milder weather conditions were recorded, the concentration of 15 phenolic compounds decreased. Further, during the third year, when weather conditions were drier than in the second but milder than the first, concentrations increased in most of the components identified. It is also interesting to remark that in 2020, after this dry and warm episode, some phenols were found at even higher concentrations than in the first year.

Notably, after falling in the second year, rosmarinic acid showed substantial increases in content in the third year, especially in Sa1. Similar behavior was observed for several other phenolic acids, namely salvianic, caffeic, and lithospermic acids, as well as salvianolic acids A and C. On the other hand, while marked decreases in concentration were observed from the first to the second year in most flavonoids (including luteolin-7-O-rutinoside, luteoilin-7-O-glucuronide, luteolin-4-O-glucoside, luteolin, hispidulin, cirsimaritin, genkwanin, and salvigenin), the apigenin derivative measured (apigenin-7-O-glucoside) showed a different pattern, with a pronounced increase from the first to the second year, and a further increase in the third.

### 2.4. Antioxidant Activity

**Figure 2:**
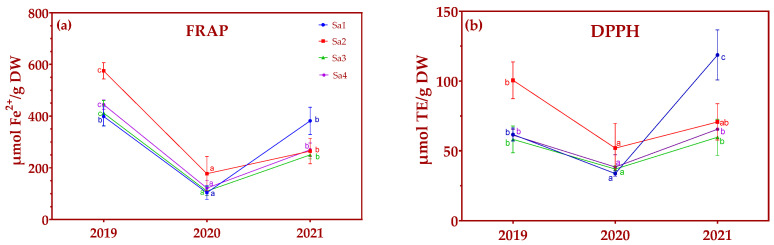
Antioxidant capacity of *Salvia lavandulifolia* Vahl. in four ecotypes (Sa1–Sa4) over three consecutive crop years as assessed by (**a**) FRAP assay and (**b**) DPPH assay. Different letters indicate a significant difference between years at *p* < 0.05.

In most ecotypes, the antioxidant activity measured over three consecutive years of cultivation showed a marked reduction in adult plants (second crop year) compared to young plants (first crop year) in the FRAP assay (Figure 2a). In addition, it can be observed that though FRAP activity was highest in Sa2 in the first year (575.2 ± 31.35 µmol Fe^2+^/g DW), as in other ecotypes, it then fell sharply in the second year, and despite recovering somewhat by the end of the experiment, did not reach baseline levels (264.9 ± 49.08 µmol Fe^2+^/g DW). This pattern was also observed in Sa3 and Sa4. That is, these ecotypes showed high levels of activity in the first year (harsh environmental conditions), activity decreasing in the second year (milder conditions), and then increasing in the third year, but without reaching the initial activity levels.

In the case of the DPPH assay (Figure 2b), the behavior observed was similar to that shown by FRAP, with all ecotypes showing less activity in the second year of cultivation followed by a recovery in the third year. Nevertheless, in Sa1, the antioxidant capacity increased to above that in the other ecotypes in the third year, almost doubling the values in the first year (118.7 ± 34.77 and 61.7 ± 4.64 µmol TE/g DW, respectively).

To evaluate the relationships between the quantitative phenolic profile and the corresponding antioxidant capacity, correlation coefficients were calculated (Table 3). Results showed a strong correlation (*p* < 0.01) between DPPH and FRAP in vitro antioxidant activity and the content of the following phenols: lithospermic acid, luteolin-4-O-glucoside, rosmarinic acid, and salvianolic acid A. Further, salvianic acid and caffeic acid levels were strongly correlated with DPPH activity, and luteolin-7-O-glucuronide and cirsiliol levels with FRAP reduction power.

## 3. Discussion

The adverse impacts of climate change significantly influence agricultural production, particularly that of rainfed crops. Under drought conditions, plants adapt by decreasing stomatal density and leaf size, thus minimizing water loss and maintaining internal water balance [20]. With a reduction in photosynthetic materials, there is a corresponding decrease in EO yield, as it depends on the percentage of EO and the yield of the aerial part, which is negatively affected when the plant is under stress [21]. In line with this, it has been reported that the *Salvia* genus exhibits high variability in EO yield and chemotype, which could be related to environmental conditions as well as genetic factors [22]. In this context, more data are needed to help understand the agronomic behavior of this economically important rainfed crop over time under the harsh climatic conditions typical of southeast Spain and to help improve and standardize its production. Notably, our evaluation of four ecotypes over three years of cultivation shows that Spanish sage may reach the maximum yield of biomass production in the second year of cultivation. This statement is based on the fact that despite the amount of rainfall at the study site being higher in 2020 than in 2021, no significant changes were detected in biomass production between these years in any of the four ecotypes. It may be speculated that, once the plants reach their adult size, they barely modify biomass production over time. Nonetheless, more studies, over a longer period, are needed to confirm this.

A similar conclusion was also reached by Garcia–Caparros [18], who reported no effects of drought stress on biomass production for *Salvia lavandulifolia*. Additionally, it is known that biomass production is directly related to EO yield per hectare [23]. Interestingly, in our study, changes in EO yield (Figure 1) did not reflect the levels of biomass produced. Previous studies conducted by Usano et al., [2] concerning *Salvia lavandulifolia* EO yield and quality over four years of cultivation, found that the EO yield increased progressively from the second to the fourth year of cultivation. On the other hand, Sánchez-Vioque et al. [7] reported that the year of cultivation had a minimal effect on the biomass and EO production for this species. According to our results, the pattern of biomass and EO yield over time is ecotype dependent, since among the four ecotypes studied, two of them (Sa1 and Sa4) showed increases in biomass and EO production over time, while decreases in production were observed in the other two (Sa2 and Sa3) from the second to the third year of cultivation. Taking these results into account and considering that no direct proportionality was found between biomass production and EO yield, it could be speculated that the synthesis of EO by these species is genetically predetermined. This statement supports the hypothesis of Usano et al. [24] that genetic heritage influences EO yield and composition, which is based on their observation of intraspecific variability within populations when they cultivated clones from different wild Spanish sage ecotypes under the same environmental conditions.

Regarding EO profile, as expected, the order of abundance of the components that define the chemotype was maintained after the micropropagation and establishment of the experimental crops; however, differences were found between the relative concentrations of these compounds, which confirms the effect of the edaphoclimatic conditions on the EO volatile profile. These findings are supported by previous evidence that the EO quantitative profile is modulated by environmental factors, while the qualitative composition is mainly determined by genetic heritage [7]. This last factor could explain the differences found in the volatile profile of the four ecotypes under study, since it implies that chemical variability is not only associated with the geographic origin of the plants but also with the intraspecific variability between individuals belonging to the same wild population [6,25]. In this context, experiments with clones from preselected plants seemed warranted to be able to relate real changes to external factors rather than to variability between the plants. In our study, the changes in EO volatile profile over three years of cultivation have shown that the response is ecotype dependent.

Major components defining the chemotype, camphor, 1,8-cineole, borneol, and camphene, showed different patterns of change associated with the ecotype under study. Climatic conditions showed slight variations over the three years (Table 4), and changes might be related to the levels of potential evapotranspiration of the area (ETo) and precipitation. Plants are known to accumulate phenylpropanoids and terpenoids as a response to environmental pressures. These components are crucial building blocks for the formation of the secondary cell wall, protect plants from harmful ultraviolet rays, help in scavenging reactive oxygen species, and act as signaling substances [26]

During the first year, the harshest weather conditions were recorded, with higher levels of ETo and lower rainfall. Under these conditions, Spanish sage ecotypes produced higher levels of camphor and 1,8-cineole, levels of both decreasing in the following years to different extents depending on the ecotype under study. The synthesis of other components, including borneol, monoterpene hydrocarbons, major sesquiterpenes, and terpinen-4-ol, was favored by the higher levels of precipitation during the second and third years of experimentation. Considering our results with clones of four ecotypes, and the conclusions of Sánchez-Vioque et al. [7], that genetic factors, more than environmental conditions or plant age, significantly influence EO volatile profiles, it can be stated that the EO volatile profile of Spanish sage is modulated over time depending on climatic conditions but that the range of such variations is ecotype dependent. That is, both intrinsic and extrinsic factors affect the quality of the EO produced by this species.

Concerning the phenolic fraction, extracted from the distilled plant material, previous studies conducted by Ortiz de Elguea-Culebras et al. [27] showed no differences associated with changes in the phenolic profile over time for different Spanish sage ecotypes collected in diverse locations across Spain. These authors pointed out the influence of both genetic factors and environmental conditions (related to different habitats of the origin of ecotypes) on the stability of these phenolic fractions. However, our results concerning the evolution over time of the polyphenolic profile of clones from different ecotypes collected from the same geographical area and cultivated under the same environmental conditions showed a different pattern, which could only be associated with genetic factors. Specifically, the response was ecotype dependent and an influence of weather conditions was also evident. During the second year of cultivation, the level of rainfall recorded was higher than in the first and third years of study. Under these conditions, the concentration of the major polyphenolic components was lower than in the other years of cultivation. Similarly, Trócsányi et al. [28] reported that warmer and drier conditions increased rosmarinic acid synthase gene expression levels and, in turn, the concentration of rosmarinic acid in *Thymus vulgaris*. This issue was also explored by Bistgani et al. [29], who affirmed that drought stress affects flavonoid concentrations in leaves by altering gene expression. In relation to this, Baghalian et al. [30] found a reduction in the synthesis of apigenin following a lack of watering in *Matricaria recutita*. Controversies regarding the extent of the impact of drought stress on the synthesis of polyphenols could be related to the length of stress exposure and/or the developmental stages of plants [29]. In our study, we observed a direct relationship between drought and the quantitative level of polyphenols synthesized by *Salvia lavandulifoila* Vahl.

Similarly, variations were also observed in the antioxidant activity of *Salvia lavandulifolia* methanolic extracts in our study. Young plants under hard semiarid conditions (first year of cultivation) showed potent antioxidant power, and this was related, based on correlation coefficients, to higher concentrations of lithospermic acid, luteolin-7-O-glucoside, rosmarinic acid, and salvianolic acid A. Notably, one ecotype (Sa2) had higher concentrations of these compounds in the first year of cultivation, which could explain the higher antioxidant capacities of its methanolic extracts. Previous research conducted by Semwal et al. [31] pointed out the high antioxidant potential of lithospermic acid. This phenolic acid prevents the production of superoxide radicals and lipid peroxidation, protecting plant tissue from the harmful effects of reactive oxygen species. Additionally, Gunnaiah et al. [32] pointed out that phenolic glucosides, hydroxycinnamic acid derivatives, and flavonoids are involved in thickening the secondary cell wall, which makes plants more drought tolerant. This cell reinforcement, together with their chemical functions, suggests that these phenolic components play an important role in plant resistance to oxidative stress [33]. These statements support the hypothesis that, under harsh drought conditions, *Salvia lavandulifolia* increases the synthesis of several phenolic components (lithospermic acid, luteolin-7-O-glucoside, rosmarinic acid, and salvianolic acid A). Further research is warranted to confirm these findings and to explore their implications for the cultivation of Spanish sage under changing climatic conditions.

## 4. Materials and Methods

### 4.1. Crop Experimental Design and Plant Material

This experimental assay was conducted at “Chaparral” (38°06′39.6″ N 1°40′50.0″ W, 400 m above sea level), an experimental station of the IMIDA in Murcia (Spain). The top 30 cm of the growing area consists of 38% silt, 30% sand, and 33% clay. The saturation percentage of the soil is 36%. It is alkaline, with a pH of 8.07, and it has an electrical conductivity of 0.85 dS/m. The semiarid climatic conditions of the local area (annual average rainfall, temperature, and evapotranspiration) during the experiment are summarized in Table 4.

For the development of this assay, four Spanish sage ecotypes, coming from the IMIDA germplasm bank, were preselected based on their essential oil yield (% *v*/*w)* and chemotype (% relative quantification) (Table 5).

From these plants, cloned plant material was obtained from in vitro culture following the protocol described by Cáceres-Cevallos et al. [34] with some modifications. Explants from the preselected ecotypes were sterilized in ethanolic solution (70%, *v*/*v*) for 1 min followed by washing in sodium hypochlorite (15%, *v*/*v*) for 15 min. After that, all explants were rinsed in sterilized distilled water until their establishment in Murashige and Skoog medium enriched with vitamins, sucrose (20%, *w*/*v*), and plant propagation agar (0.8%, *w*/*v*), and the pH was adjusted to 5.6–5.8. This medium was called MS and all reagents were supplied by Duchefa Biochemie (Haarlem, The Netherlands).

After 1 month, all explants were transferred to a multiplication medium that consisted of MS enriched with 6-benzylaminopurine (0.5 mg.L^−1^) and gibberellic acid (0.5 mg.L^−1^) for 2 months. Then, all multiplied explants were placed into a rooting medium comprised of MS media enriched with kinetin (0.1 mg.L^−1^) and indole-3-acetic acid (0.1 mg.L^−1^) for 2 months. Finally, all plantlets were acclimatized and grown under greenhouse conditions for 3 months until transplantation to the experimental area in April 2018.

A trial was designed with three randomized blocks and ten experimental replications per block and ecotype. Each replication area was 10 m^2^ with a density of 200 plants/100 m^2^.

This rainfed crop requires minimal nutrients and just received a base dressing before planting (10 tn of sheep manure/ha). Weed control was managed mechanically between rows and manually on the rows from the first year of planting.

To evaluate the agronomic yield over three years of cultivation, three consecutive harvests were made during the first half of July in 2019, 2020, and 2021, when the plant material showed a phenological stage of full blooms and the beginning of fructification. The freshly harvested plant material was immediately weighed, and a 3-kg sample was taken to assess the production of dry matter (DM). To measure the DM per 100 m^2^ of cultivation area, the plant material was dried in a forced-air dryer at 35 °C for 48 h until it reached a constant weight. The DM weight obtained from 3 kg of fresh plant material was extrapolated to the total production in 100 m^2^ of cultivation area.

### 4.2. Essential Oil Extraction

Dry plant material was hydro-distilled following the protocol described in the 10th edition of the European Pharmacopoeia [35] using a Clevenger apparatus. The essential oil obtained was dried with anhydrous sodium sulfate and kept in amber vials at 5 °C until their chromatographic analysis.

Agronomic yield was measured as kg of fresh weight per hectare (kg FW.ha^−1^) and the essential oil yield was estimated as the volume of essential oil per hectare (EO.ha^−1^).

### 4.3. Gas Chromatography-Mass Spectrometry Analysis

Gas chromatography-mass spectrometry (GC-MS) analysis was performed following the method described by Farhat et al. [36]. In brief, essential oil samples (0.1 µL) were subjected to analysis by GC-MS using a 6890 N gas chromatograph (Agilent Technologies, Palo Alto, CA, USA) equipped with an HP-5MS UI column (30 m × 0.25 mm, 0.25 µm, Agilent Technologies). Helium was used as the carrier gas (constant pressure, β-ionone eluting at 27.6 min), and the split ratio was set to 100:1. The gas chromatograph was linked to an Agilent 5972 inert mass-selective detector. The initial oven temperature was set at 60 °C, then increased at 2.5 °C.min^−1^ to 155 °C, and finally raised to 250 °C at a rate of 10 °C.min^−1^. The injection port and transfer line to the mass selective detector were kept at 250 °C and 280 °C, respectively. The mass spectrometer was operated in electron impact ionization mode with an ionizing energy of 70 eV, covering a mass-to-charge ratio from 50 to 500 at 3.21 scan.s^−1^. The quadrupole temperature was 150 °C, and the electron multiplier voltage was maintained at 1300 V.

The individual peaks were identified by retention times and retention indices (relative to C6-C17 n-alkanes), compared with those of known compounds (Sigma-Aldrich, and by comparison of mass spectra using the NBS75K library (U.S. National Bureau of Standards, 2002) and spectra obtained from the standard. Non-isothermal Kovats retention indices were calculated using the definition of Van den Dool and Kratz [37].

Ix = 100n + 100(tx − tn)/(tn+1 − tn)

where tn and tn+1 are retention times of the reference n-alkane hydrocarbons eluting immediately before and after the chemical compound; and tx is the retention time of compound X.

Percentage compositions of samples were measured according to the area of the chromatographic peaks using the total ion current.

### 4.4. Extraction of Polyphenolic Compounds

Residual plant material from the distillation process was dried in a forced-air dryer at 35 °C for 48 h to reach a constant weight, and then ground to pass through a 2-mm mesh. Polyphenolic compounds were extracted following the method described by Jordán et al. [38]. In brief, 500 mg of dried samples were extracted using 150 mL of methanol in a Soxhlet extractor (B-811) (Buchi, Flawil, Switzerland) for 2 h under a nitrogen atmosphere. Methanolic extracts (ME) were taken to dryness at 40 °C under vacuum conditions in an evaporator system (Syncore Polyvap R-96) (Buchi, Flawil, Switzerland). The residue was re-dissolved in methanol and made up to 5 mL. The yield of the extracts was expressed in terms of milligrams of dry ME per gram of dry plant weight. Final extracts were kept in vials at −80 °C until the qualitative analysis by HPLC-DAD.

### 4.5. HPLC-DAD Qualitative Analyses

High-performance liquid chromatography (HPLC) analysis was carried out, following the method described by Jordán et al. [39], on a reverse phase Zorbax SB-C18 column (4.6 mm × 250 mm, 5 µm pore size, Agilent Technologies, USA) with a guard column (Zorbax SB-C18 4.6 mm × 125 mm, 5 µm pore size, Agilent Technologies, USA) at ambient temperature. Extracts were filtered with a 0.45-µm filter (Millipore SAS, Molsheim, France) and 20 µL was injected into an Agilent 1200 series HPLC system (Agilent Technologies, Santa Clara, CA, USA) equipped with a vacuum degasser, auto-sampler, quaternary pump, and diode-array detection (DAD).

The mobile phase was acetonitrile (B) and acidified water containing 0.05% formic acid (A). The gradient used was: 0 min, 5% B; 10 min, 15% B; 30 min, 25% B; 35 min, 30% B; 50 min, 55% B; 55 min, 90% B; 57 min, and 100% B, held for 10 min before returning to the initial conditions. The flow rate was 1.0 mL.min^−1^, and the wavelengths of detection were set at 280 and 330 nm. To quantify the results, linear regression models were determined using standard dilution techniques. Phenolic compound contents were expressed as milligrams per gram of dry plant.

### 4.6. Antioxidant Activity

Antioxidant activity was assessed using ferric-reducing antioxidant power (FRAP) and 2,2-diphenyl-1-picrylhydrazyl (DPPH) methods. In both cases, we used techniques described by Jordan et al. [40] with some modifications.

#### 4.6.1. Ferric Reducing Antioxidant Power (FRAP)

FRAP was measured as the ability of the methanolic extract to reduce ferric ions following the method described by Benzie and Strain [41]. In brief, FRAP reagent was made with 10 mM 2,4,6-tripyridyl-S-triazine prepared with 40 mM HCl, 300 mM buffer acetate (pH 3.6), and 20 mM FeCl3. The three solutions were mixed at a ratio of 10:1:1 (by volume). A 40-μL aliquot was combined with 1.2 mL of FRAP reagent and the mixture was incubated at 37 °C for 30 min in darkness. All samples were measured spectrophotometrically at 593 nm. For the calibration curve, we used working solutions with known concentrations of Fe (II) (FeSO_4_7H_2_O) ranging from 0.1 to 1 mM. Results were expressed as micromoles of FeSO_4_ × 7H_2_O.g^−1^ of dry plant.

#### 4.6.2. DPPH^•^ Radical-Scavenging Activity (DPPH)

To evaluate the ability of methanolic extracts to scavenge DPPH^•^ free radicals, we used a method described by Brand-Williams et al. [42]. For this, 500 μL of aliquots were added to 1 mL of methanolic solution of DPPH^•^ (0.1 mM). The samples were incubated at room temperature for 20 min in darkness and then measured spectrophotometrically at 517 nm. Absorbance was measured against a blank of 500 μL of methanol mixed with 1 mL of DPPH^•^. The calibration curve of Trolox used ranged from 100 to 450 µM. Results were expressed as µmoles of Trolox equivalents.g^−1^ of dry plant.

### 4.7. Statistical Analyses

Data are shown as average ± SE. Results were analyzed by one-way analysis of variance (ANOVA), conducting Levene’s test to verify equality of variances. Fisher’s least significant difference test was used to compare means at *p* < 0.05. Correlations between variables were measured by Pearson’s correlation coefficients. All statistical analyses were performed with STATGRAPHICS Centurion XVI.I.

## 5. Conclusions

Growth of clones from four ecotypes, under the same edaphoclimatic conditions, indicates that Spanish sage reaches a stable biomass production in the second year of cultivation. Changes in essential oil yield over time were ecotype dependent. Considering biomass production and essential oil yield, it is important to highlight that an increase in biomass did not always translate to an increase in essential oil yield. This finding supports the view that essential oil production may be genetically predetermined. Regarding the evolution of secondary metabolism in this species, to overcome the negative effect under the extremely harsh environmental conditions studied, Spanish sage ecotypes seem to have produced higher levels of camphor and 1,8-cineole in its essential oil, along with lithospermic acid, luteolin-7-O-glucoside, rosmarinic acid, and salvianolic acid A in their nonvolatile fraction. In turn, this increase in the synthesis of these polyphenolic compounds leads to greater antioxidant activity which might protect the plants from the aforementioned types of abiotic stress. In conclusion, the secondary metabolism of Spanish sage seems to be influenced by weather conditions over time, with the extent of variation dependent on the ecotype. Both intrinsic and extrinsic factors similarly impact the production quality of this species. Especially in the context of climate change, understanding this agronomic behavior is crucial for the selection of elite ecotypes that will allow further genetic improvement programs for *Salvia lavandulifolia* Vahl.

## Figures and Tables

**Table 1:** Essential oil volatile profile of four ecotypes (Sa1–Sa4) of *Salvia lavandulifolia* over three years of cultivation.

Component Relative Concentration (%)	RI *	Year	Sa1	Sa2	Sa3	Sa4
tricyclene		2019	0.3 ± 0.04	n.d.	tr	1.7 ± 0.13 ^a^
	923	2020	0.3 ± 0.05	0.1 ± 0.05	0.1 ± 0.00	1.7 ± 0.23 ^a^
		2021	0.3 ± 0.06	0.2 ± 0.03	0.1 ± 0.01	2.1 ± 0.01 ^b^
α-thujene		2019	0.1 ± 0.02 ^a^	0.1 ± 0.05	tr	0.1 ± 0.02
	930	2020	0.2 ± 0.05 ^b^	0.2 ± 0.03	0.2 ± 0.05	0.2 ± 0.07
		2021	0.2 ± 0.01 ^b^	0.1 ± 0.01	0.1 ± 0.03	0.2 ± 0.00
α-pinene		2019	4.9 ± 1.08 ^a^	2.4 ± 0.70	1.7 ± 0.17 ^a^	1.7 ± 0.13 ^a^
	963	2020	7.8 ± 2.21 ^b^	1.8 ± 0.95	4.3 ± 3.12 ^b^	1.7 ± 0.23 ^a^
		2021	9.1 ± 0.08 ^b^	2.7 ± 0.30	4.4 ± 1.15 ^b^	2.1 ± 0.01 ^b^
camphene		2019	9.3 ± 1.58	6.5 ± 1.38 ^b^	3.5 ± 0.45	4.1 ± 0.20
	971	2020	9.2 ± 0.60	4.7 ± 1.72 ^a^	3.7 ± 0.75	4.8 ± 0.41
		2021	10.0 ± 0.08	6.3 ± 0.62 ^ab^	4.2 ± 0.33	4.8 ± 0.16
sabinene		2019	0.2 ± 0.07	0.4 ± 0.13 ^a^	0.5 ± 0.09 ^b^	0.4 ± 0.08 ^a^
	987	2020	0.3 ± 0.09	0.2 ± 0.14 ^a^	0.3 ± 0.13 ^a^	0.4 ± 0.07 ^a^
		2021	0.4 ± 0.03	0.5 ± 0.06 ^b^	0.5 ± 0.10 ^b^	0.7 ± 0.03 ^b^
β-pinene		2019	2.5 ± 0.32 ^a^	2.5 ± 0.51 ^a^	3.3 ± 0.33 ^a^	3.1 ± 0.13 ^a^
	989	2020	6.8 ± 2.75 ^b^	4.7 ± 1.15 ^b^	6.9 ± 1.91 ^b^	5.0 ± 1.15 ^b^
		2021	5.9 ± 0.15 ^b^	4.6 ± 0.41 ^b^	5.3 ± 1.10 ^b^	3.3 ± 0.08 ^a^
myrcene		2019	2.4 ± 0.33	1.7 ± 0.28	1.2 ± 0.37	0.9 ± 0.15 ^a^
	998	2020	1.5 ± 0.95	1.2 ± 0.82	1.2 ± 0.41	1.1 ± 0.32 ^b^
		2021	2.4 ± 0.13	1.8 ± 0.29	1.1 ± 0.16	1.5 ± 0.04 ^c^
α-terpinene		2019	0.1 ± 0.02	tr	0.1 ± 0.22	tr
	1017	2020	0.1 ± 0.01	0.1 ± 0.01	0.1 ± 0.01	0.2 ± 0.05
		2021	0.1 ± 0.08	0.1 ± 0.00	0.1 ± 0.01	0.2 ± 0.01
*p*-cymene		2019	0.1 ± 0.03	0.1 ± 0.03 ^a^	0.1 ± 0.02 ^a^	0.3 ± 0.09 ^a^
	1024	2020	0.1 ± 0.03	0.5 ± 0.03 ^c^	0.4 ± 0.06 ^b^	0.8 ± 0.15 ^c^
		2021	0.1 ± 0.00	0.3 ± 0.05 ^b^	0.3 ± 0.07 ^b^	0.5 ± 0.00 ^b^
limonene		2019	4.5 ± 0.71 ^b^	4.5 ± 0.5 ^b^	4.5 ± 0.24 ^b^	4.7 ± 0.14 ^a^
	1027	2020	3.6 ± 0.44 ^a^	2.8 ± 0.69 ^a^	3.9 ± 0.53 ^a^	6.3 ± 0.77 ^b^
		2021	4.7 ± 0.09 ^ab^	4.0 ± 0.29 ^ab^	3.9 ± 0.29 ^a^	4.7 ± 0.34 ^a^
1,8-cineole		2019	2.8 ± 0.47 ^b^	21.1 ± 2.40 ^c^	32.3 ± 6.76 ^c^	34.0 ± 1.05 ^c^
	1030	2020	1.8 ± 1.03 ^a^	3.9 ± 1.98 ^a^	9.9 ± 1.40 ^a^	17.9 ± 4.04 ^a^
		2021	2.5 ± 0.05 ^ab^	8.7 ± 1.88 ^b^	14.9 ± 2.25 ^b^	26.0 ± 1.13 ^b^
*(Z)-*β-ocimene		2019	n.d.	0.7 ± 0.14 ^a^	tr	0.3 ± 0.04
	1038	2020	n.d.	0.2 ± 0.06 ^b^	0.1 ± 0.00	0.2 ± 0.06
		2021	n.d.	1.5 ± 0.07 ^c^	0.1 ± 0.03	0.8 ± 0.03
*(E)-*β-ocimene		2019	n.d.	0.1 ± 0.02 ^a^	0.04 ± 0.01 ^a^	0.1 ± 0.1 ^a^
	1048	2020	n.d.	1.1 ± 0.29 ^b^	0.1 ± 0.00 ^b^	0.9 ± 0.31 ^b^
		2021	n.d.	0.4 ± 0.01 ^c^	0.2 ± 0.05 ^b^	0.2 ± 0.00 ^b^
γ-terpinene		2019	0.1 ± 0.04 ^a^	0.2 ± 0.02 ^a^	0.2 ± 0.05 ^a^	0.4 ± 0.14 ^a^
	1053	2020	0.2 ± 0.02 ^b^	0.5 ± 0.39 ^b^	0.5 ± 0.24 ^b^	1.5 ± 0.59 ^c^
		2021	0.3 ± 0.00 ^b^	0.7 ± 0.09 ^b^	0.6 ± 0.09 ^b^	1.0 ± 0.06 ^b^
*(Z)*-sabinene hydrate		2019	0.1 ± 0.04	0.1 ± 0.02	0.1 ± 0.03	0.2 ± 0.07
	1061	2020	0.1 ± 0.05	0.1 ± 0.03	0.2 ± 0.03	0.3 ± 0.09
		2021	0.1 ± 0.01	0.2 ± 0.01	0.2 ± 0.01	0.3 ± 0.06
α-terpinolene		2019	0.4 ± 0.08	0.2 ± 0.06 ^a^	0.2 ± 0.06 ^a^	0.3 ± 0.06 ^a^
	1080	2020	0.4 ± 0.04	0.41 ± 0.15 ^b^	0.4 ± 0.08 ^b^	0.6 ± 0.08 ^b^
		2021	0.7 ± 0.02	0.6 ± 0.02 ^c^	0.5 ± 0.02 ^c^	0.7 ± 0.01 ^c^
linalool		2020	0.4 ± 0.10	tr	4.4 ± 1.39 ^b^	tr
	1094	2021	0.4 ± 0.06	0.1 ± 0.03	2.8 ± 1.29 ^a^	0.1 ± 0.02
		2022	0.7 ± 0.08	0.2 ± 0.02	4.6 ± 0.83 ^ab^	0.2 ± 0.00
camphor		2019	43.9 ± 4.11 ^c^	42.4 ± 1.82 ^b^	31.5 ± 2.21 ^b^	44.6 ± 1.22 ^b^
	1136	2020	35.1 ± 2.28 ^b^	29.6 ± 9.45 ^a^	26.1 ± 2.62 ^a^	42.2 ± 4.69 ^ab^
		2021	30.4 ± 1.02 ^a^	30.5 ± 0.54 ^a^	25.8 ± 1.13 ^a^	39.2 ± 0.93 ^a^
borneol		2019	12.7 ± 4.44	9.1 ± 1.84	4.1 ± 0.74 ^a^	1.5 ± 0.07 ^a^
	1159	2020	15.8 ± 2.69	11.3 ± 2.77	17.0 ± 3.74 ^c^	4.6 ± 1.06 ^c^
		2021	10.0 ± 0.91	10.0 ± 0.75	10.9 ± 2.63 ^b^	3.3 ± 0.31 ^b^
γ- terpineol		2019	tr	0.4 ± 0.11 ^b^	0.5 ± 0.06 ^b^	0.4 ± 0.07
	1161	2020	n.d.	n.d.	n.d.	0.4 ± 0.08
		2021	0.1 ± 0.08	0.1 ± 0.01 ^a^	0.3 ± 0.06 ^a^	0.5 ± 0.02
terpinen-4-ol		2019	0.5 ± 0.09	0.4 ± 0.1 ^a^	0.3 ± 0.05 ^a^	0.2 ± 0.06 ^a^
	1172	2020	0.5 ± 0.04	0.4 ± 0.09 ^a^	0.5 ± 0.05 ^b^	0.4 ± 0.07 ^b^
		2021	0.7 ± 0.02	0.6 ± 0.00 ^b^	0.6 ± 0.03 ^c^	0.6 ± 0.03 ^c^
α-terpineol		2019	0.1 ± 0.04 ^a^	0.1 ± 0.03 ^a^	1.1 ± 0.19 ^b^	1.1 ± 0.19 ^b^
	1187	2020	0.2 ± 0.04 ^a^	0.1 ± 0.02 ^a^	0.8 ± 0.29 ^a^	0.7 ± 0.06 ^a^
		2021	0.3 ± 0.01 ^b^	0.2 ± 0.02 ^b^	1.4 ± 0.34 ^b^	1.2 ± 0.08 ^b^
nerol		2019	0.2 ± 0.06	n.d.	0.6 ± 0.22 ^a^	tr
	1229	2020	0.3 ± 0.01	n.d.	n.d.	n.d.
		2021	0.1 ± 0.13	0.1 ± 0.02	1.0 ± 0.08 ^a^	0.3 ± 0.02
bergamol/linalyl acetate		2019	0.4 ± 0.15	n.d.	5.6 ± 0.71 ^b^	n.d.
	1255	2020	0.4 ± 0.06	n.d.	2.8 ± 1.20 ^a^	0.1 ± 0.06
		2021	0.2 ± 0.23	n.d.	4.7 ± 1.25 ^b^	n.d.
bornyl acetate		2019	5.1 ± 1.09	1.7 ± 0.5	1.9 ± 0.47 ^a^	0.5 ± 0.37 ^a^
	1284	2020	3.9 ± 1.94	1.6 ± 0.36	3.2 ± 0.81 ^b^	1.3 ± 0.56 ^b^
		2021	3.3 ± 4.66	1.5 ± 0.06	2.3 ± 0.06 ^a^	1.7 ± 0.17 ^b^
α-terpinyl acetate		2019	0.1 ± 0.06 ^a^	n.d.	1.6 ± 0.23 ^b^	n.d.
	1302	2020	0.2 ± 0.06 ^b^	n.d.	0.8 ± 0.23 ^a^	n.d.
		2021	0.2 ± 0.04 ^b^	n.d.	1.5 ± 0.30 ^b^	n.d.
isobornyl propionate		2019	0.3 ± 0.13 ^b^	n.d.	n.d.	n.d.
	1376	2020	0.1 ± 0.05 ^a^	0.1 ± 0.03	0.3 ± 0.08	0.1 ± 0.03
		2021	0.2 ± 0.02 ^ab^	0.1 ± 0.01	0.3 ± 0.04	0.1 ± 0.02
geranyl acetate		2019	0.1 ± 0.05	n.d.	0.4 ± 0.12 ^a^	n.d.
	1380	2020	0.1 ± 0.07	n.d.	0.3 ± 0.09 ^a^	n.d.
		2021	0.2 ± 0.02	n.d.	0.7 ± 0.21 ^b^	0.1 ± 0.01
β-caryophyllene		2019	1.5 ± 0.67 ^a^	0.2 ± 0.11 ^a^	tr ^a^	0.2 ± 0.11 ^a^
	1423	2020	4.2 ± 1.31 ^b^	2.3 ± 0.40 ^b^	1.6 ± 0.43 ^a^	2.7 ± 0.47 ^c^
		2021	5.3 ± 0.01 ^b^	2.8 ± 0.29 ^c^	1.3 ± 0.33 ^b^	1.4 ± 0.10 ^b^
α-humulene		2019	0.04 ± 0.02	0.1 ± 0.02 ^a^	0.1 ± 0.06 ^a^	n.d.
	1453	2020	0.1 ± 0.03	1.2 ± 0.23 ^b^	1.7 ± 0.48 ^c^	0.1 ± 0.02
		2021	0.2 ± 0.00	1.3 ± 0.16 ^b^	1.3 ± 0.33 ^b^	0.1 ± 0.01
geranyl propanoate		2019	0.2 ± 0.09	n.d.	0.5 ± 0.10 ^b^	0.7 ± 0.34 ^a^
	1477	2020	n.d.	0.1 ± 0.02	0.2 ± 0.04 ^a^	0.5 ± 0.26 ^a^
		2021	0.2 ± 0.07	0.1 ± 0.03	0.4 ± 0.18 ^b^	1.4 ± 0.24 ^b^
γ-elemene		2019	0.1 ± 0.04	0.2 ± 0.10 ^a^	n.d.	n.d.
	1480	2020	n.d.	1.2 ± 0.32 ^b^	0.2 ± 0.09	0.8 ± 0.23
		2021	0.1 ± 0.07	2.1 ± 0.23 ^c^	0.1 ± 0.02	0.6 ± 0.07
λ-cadinene		2019	0.3 ± 0.15	tr	n.d.	n.d.
	1523	2020	0.2 ± 0.18	0.4 ± 0.22	0.5 ± 0.28	0.2 ± 0.06 ^b^
		2021	0.5 ± 0.01	0.5 ± 0.06	0.4 ± 0.02	0.1 ± 0.00 ^a^
spathulenol		2019	0.3 ± 0.22	0.5 ± 0.5 ^a^	n.d.	n.d.
	1576	2020	0.3 ± 0.05	4.5 ± 2.02 ^c^	1.1 ± 0.62 ^b^	0.8 ± 0.23 ^b^
		2021	0.2 ± 0.02	2.6 ± 0.39 ^b^	0.2 ± 0.00 ^a^	0.2 ± 0.04 ^a^
caryophyllene oxide		2019	1.4 ± 1.05	0.2 ± 0.30 ^a^	n.d.	tr
	1581	2020	1.9 ± 0.28	2.4 ± 1.06 ^c^	1.1 ± 0.89	0.6 ± 0.16 ^b^
		2021	1.5 ± 0.10	1.1 ± 0.25 ^b^	0.4 ± 0.14	0.2 ± 0.03 ^a^
*Ζ*-cadinol		2019	n.d.	n.d.	n.d.	n.d.
	1652	2020	0.3 ± 0.40	0.2 ± 0.08 ^b^	0.8 ± 0.65	0.2 ± 0.08 ^b^
		2021	0.4 ± 0.04	0.1 ± 0.01 ^a^	0.3 ± 0.03	0.1 ± 0.01 ^a^

n.d.: not detected; tr: traces (<0.1); For each component, different letters indicate a significant difference between years at *p* < 0.05 * Retention indices calculated using a non-polar column (HP-5).

**Table 2:** Phenolic profile analysis of four *Salvia lavandulifolia* Vahl. ecotypes (Sa1–Sa4).

Phenolic Compounds mg.g DW^−1^	Year	Sa1	Sa2	Sa3	Sa4
Salvianic acid	2019	0.51 ± 0.04 ^b^	0.95 ± 0.14 ^b^	0.68 ± 0.07 ^b^	0.68 ± 0.03 ^b^
	2020	0.21 ± 0.03 ^a^	0.70 ± 0.08 ^a^	0.26 ± 0.03 ^a^	0.25 ± 0.04 ^a^
	2021	1.05 ± 0.09 ^c^	1.03 ± 0.07 ^b^	1.03 ± 0.08 ^c^	0.95 ± 0.09 c
Caffeic acid	2019	0.14 ± 0.01 ^b^	0.18 ± 0.03 ^b^	0.16 ± 0.03 ^b^	0.19 ± 0.01 ^b^
	2020	0.12 ± 0.02 ^a^	0.13 ± 0.08 ^a^	0.11 ± 0.01 ^a^	0.09 ± 0.02 ^a^
	2021	0.29 ± 0.03 ^c^	0.23 ± 0.04 ^b^	0.26 ± 0.01 ^c^	0.24 ± 0.02 ^c^
Luteolin-7-O-rutinoside	2019	n.d.	n.d.	n.d.	n.d.
	2020	0.05 ± 0.01 ^a^	0.03 ± 0.08 ^a^	0.04 ± 0.01 ^a^	0.03 ± 0.01 ^a^
	2021	0.17 ± 0.02 ^b^	0.10 ± 0.02 ^b^	0.11 ± 0.02 ^b^	0.07 ± 0.01 ^b^
Luteolin-7-O-glucuronide	2019	1.13 ± 0.10 ^c^	1.28 ± 0.09 ^b^	1.06 ± 0.08 ^c^	0.79 ± 0.03 ^c^
	2020	0.46 ± 0.06 ^a^	0.52 ± 0.08 ^a^	0.41 ± 0.08 ^a^	0.30 ± 0.04 ^a^
	2021	0.84 ± 0.10 ^b^	0.58 ± 0.11 ^a^	0.58 ± 0.05 ^b^	0.49 ± 0.04 ^b^
Apigenin-7-O-glucoside	2019	0.21 ± 0.02 ^a^	0.38 ± 0.03 ^b^	0.14 ± 0.01 ^b^	0.12 ± 0.01 ^c^
	2020	0.22 ± 0.06 ^a^	0.47 ± 0.08 ^a^	0.24 ± 0.07 ^a^	0.09 ± 0.01 ^b^
	2021	0.97 ± 0.13 ^b^	0.63 ± 0.08 ^c^	0.54 ± 0.10 ^c^	n.d.^a^
Luteolin-4-O-glucoside	2019	0.41 ± 0.04 ^b^	0.55 ± 0.06 ^b^	0.29 ± 0.02 ^c^	0.26 ± 0.02 ^c^
	2020	0.12 ± 0.02 ^a^	0.33 ± 0.08 ^a^	0.09 ± 0.01 ^a^	0.07 ± 0.01 ^a^
	2021	0.53 ± 0.07 ^c^	0.26 ± 0.05 ^a^	0.18 ± 0.02 ^b^	0.21 ± 0.02 ^b^
Rosmarinic acid	2019	2.10 ± 0.10 ^b^	2.47 ± 0.22 ^c^	1.86 ± 0.21 ^c^	2.02 ± 0.11 ^b^
	2020	0.89 ± 0.11 ^a^	2.09 ± 0.08 ^b^	0.90 ± 0.12 ^a^	0.96 ± 0.10 ^a^
	2021	3.77 ± 0.49 ^c^	1.43 ± 0.21 ^a^	1.56 ± 0.10 ^b^	2.70 ± 0.22 ^c^
Lithospermic acid	2019	0.22 ± 0.01 ^b^	0.37 ± 0.04 ^c^	0.24 ± 0.05 ^b^	0.22 ± 0.01 ^c^
	2020	0.06 ± 0.01 ^a^	0.14 ± 0.08 ^a^	0.08 ± 0.01 ^a^	0.08 ± 0.02 ^a^
	2021	0.34 ± 0.02 ^c^	0.24 ± 0.05 ^b^	0.22 ± 0.03 ^b^	0.16 ± 0.02 ^b^
Salvianolic acid A	2019	2.10 ± 0.07 ^c^	4.03 ± 0.21 ^c^	1.03 ± 0.05 ^b^	1.16 ± 0.06 ^b^
	2020	0.44 ± 0.05 ^a^	2.91 ± 0.08 ^b^	0.69 ± 0.10 ^a^	0.28 ± 0.07 ^a^
	2021	1.73 ± 0.28 ^b^	1.85 ± 0.25 ^a^	0.92 ± 0.12 ^b^	1.16 ± 0.17 ^b^
Salvianolic acid C	2019	0.12 ± 0.03 ^b^	0.19 ± 0.03 ^b^	0.08 ± 0.03 ^a^	0.11 ± 0.01 ^a^
	2020	0.06 ± 0.01 ^a^	0.10 ± 0.08 ^a^	0.08 ± 0.01 ^a^	0.09 ± 0.01 ^a^
	2021	0.26 ± 0.07 ^c^	0.22 ± 0.04 ^b^	0.16 ± 0.03 ^b^	0.24 ± 0.05 ^b^
Eriodictyol	2019	n.d.	n.d.	0.03 ± 0.01	0.07 ± 0.01 ^b^
	2020	0.04 ± 0.01	0.03 ± 0.08	0.03 ± 0.02	0.04 ± 0.01 ^a^
	2021	n.d.	n.d.	0.04 ± 0.00	0.05 ± 0.01 ^b^
Luteolin	2019	0.75 ± 0.08 ^b^	0.50 ± 0.02 ^c^	0.45 ± 0.03 ^c^	0.67 ± 0.02 ^c^
	2020	0.53 ± 0.03 ^a^	0.24 ± 0.08 ^a^	0.31 ± 0.04 ^a^	0.29 ± 0.05 ^a^
	2021	0.56 ± 0.09 ^a^	0.37 ± 0.02 ^b^	0.38 ± 0.02 ^b^	0.44 ± 0.05 ^b^
Apigenin	2019	0.15 ± 0.01 ^a^	0.12 ± 0.00 ^a^	0.12 ± 0.01 ^a^	0.16 ± 0.01 ^b^
	2020	0.24 ± 0.04 ^c^	0.17 ± 0.08 ^b^	0.15 ± 0.05 ^a^	0.10 ± 0.01 ^a^
	2021	0.20 ± 0.06 ^b^	0.17 ± 0.04 ^b^	0.17 ± 0.02 ^a^	0.11 ± 0.01 ^a^
Hispidulin	2019	0.51 ± 0.08 ^b^	0.22 ± 0.01 ^c^	0.24 ± 0.01 ^c^	0.52 ± 0.01 ^c^
	2020	0.09 ± 0.01 ^a^	0.05 ± 0.08 ^a^	0.07 ± 0.01 ^a^	0.09 ± 0.01 ^a^
	2021	0.15 ± 0.05 ^a^	0.08 ± 0.01 ^b^	0.12 ± 0.01 ^b^	0.26 ± 0.02 ^b^
Cirsiliol	2019	0.35 ± 0.07 ^c^	0.36 ± 0.02 ^c^	0.32 ± 0.01 ^b^	0.54 ± 0.02 ^c^
	2020	0.07 ± 0.01 ^a^	0.07 ± 0.08 ^a^	0.08 ± 0.01 ^a^	0.11 ± 0.01 ^a^
	2021	0.19 ± 0.02 ^b^	0.19 ± 0.01 ^b^	n.d.	0.32 ± 0.01 ^b^
Cirsimaritin	2019	0.46 ± 0.08 ^b^	1.01 ± 0.07 ^c^	0.85 ± 0.03 ^c^	2.70 ± 0.09 ^c^
	2020	0.09 ± 0.01 ^a^	0.15 ± 0.08 ^a^	0.15 ± 0.01 ^a^	0.46 ± 0.05 ^a^
	2021	0.15 ± 0.04 ^a^	0.25 ± 0.02 ^b^	0.24 ± 0.04 ^b^	1.11 ± 0.07 ^b^
Eupatorin	2019	0.34 ± 0.04 ^b^	0.14 ± 0.01 ^b^	0.22 ± 0.01 ^b^	0.25 ± 0.01 ^b^
	2020	0.10 ± 0.01 ^a^	0.06 ± 0.08 ^a^	0.08 ± 0.01 ^a^	0.10 ± 0.01 ^a^
	2021	n.d.	n.d.	n.d.	0.30 ± 0.02 ^c^
Genkwanin	2019	0.15 ± 0.02 ^b^	0.19 ± 0.01 ^c^	0.23 ± 0.01 ^c^	0.33 ± 0.01 ^c^
	2020	0.03 ± 0.01 ^a^	0.03 ± 0.08 ^a^	0.04 ± 0.01 ^a^	0.07 ± 0.01 ^a^
	2021	0.04 ± 0.01 ^a^	0.08 ± 0.01 ^b^	0.09 ± 0.02 ^b^	0.18 ± 0.01 ^b^
Salvigenin	2019	2.06 ± 0.31 ^c^	1.12 ± 0.07 ^c^	2.23 ± 0.09 ^c^	2.36 ± 0.06 ^c^
	2020	0.45 ± 0.03 ^a^	0.27 ± 0.08 ^a^	0.44 ± 0.01 ^a^	0.71 ± 0.06 ^a^
	2021	0.88 ± 0.13 ^b^	0.56 ± 0.03 ^b^	0.80 ± 0.10 ^b^	1.56 ± 0.06 ^b^

Results are expressed as mean ± standard deviation; n.d.: not detected. For each component, different letters indicate a significant difference between years at *p* < 0.05.

**Table 3:** Linear correlation coefficients of individual phenolic compounds versus the antioxidant activity as measured by 2,2-diphenyl-1-picrylhydrazyl (DPPH) and ferric-reducing antioxidant power (FRAP) assays.

	DPPH	FRAP
	r	
Salvianic acid	0.7459	n.s.
Caffeic acid	0.7373	n.s.
Luteolin-7-O-glucuronide	n.s.	0.8534
Luteolin-4-O-glucoside	0.8395	0.8560
Rosmarinic acid	0.8937	0.7078
Lithospermic acid	0.8307	0.8984
Salvianolic acid A	0.6783	0.7546
Cirsiliol	n.s.	0.7534

Significant correlation at *p* < 0.01; n.s. no significant correlation.

**Table 4:** Climatic data from the “El Chaparral” experimental station for the three consecutive years of the study.

Annual Average of Edaphoclimatic Conditions			
	July 2018/June 2019	July 2019/June 2020	July 2020/June 2021
ETo	760 mm	727 mm	713 mm
Precipitation	329 mm	528 mm	396 mm
Temperature	14.8 °C	14.9 °C	15.5 °C

**Table 5:** Essential oil yield and major volatile components of the four sage ecotypes (Sa1–Sa4) preselected in the wild.

	Sa1	Sa2	Sa3	Sa4
Essential oil yield (%)	3.2	4.5	5.4	10.8
Chemotype	C/Cph/α-p	C/1,8-C/α-p/Cph	1,8-C/C	C/1,8-C
Components (%)				
α-pinene	8	12	2	2
camphene	12	8	3	5
β-pinene	3	4	4	3
myrcene	4	4	2	1
limonene	6	4	2	4
1,8-cineole	5	21	40	28
camphor	40	32	17	40
linalool	1	0.1	3	0.1
borneol	4	4	1	3
linalyl acetate	-	-	5	-
bornyl acetate	5	3	1	1
terpenyl acetate	0.2	0.1	4	-

C: camphor; Cph: camphene; α-p: α-pinene; 1,8-C: 1,8-Cineole.

## Data Availability

Data are available within this article.
